# Green Tea (*Camellia sinensis*) Extract Increased Topoisomerase II*β*, Improved Antioxidant Defense, and Attenuated Cardiac Remodeling in an Acute Doxorubicin Toxicity Model

**DOI:** 10.1155/2021/8898919

**Published:** 2021-05-05

**Authors:** Pamela N. Modesto, Bertha F. Polegato, Priscila P. dos Santos, Leticia D. V. Grassi, Leticia C. C. Molina, Silmeia G. Z. Bazan, Elenize J. Pereira, Ana Angelica H. Fernandes, Alexandre T. Fabro, Vickeline N. Androcioli, Meliza G. Roscani, Sergio A. R. de Paiva, Leonardo A. M. Zornoff, Marcos F. Minicucci, Paula S. Azevedo

**Affiliations:** ^1^Internal Medicine Department, Botucatu Medical School, São Paulo State University (UNESP), Botucatu, Brazil; ^2^Department of Chemical and Biological Sciences, São Paulo State University (UNESP), Botucatu, Brazil; ^3^Department of Pathology and Legal Medicine, Ribeirão Preto Medical School, University of São Paulo (UNESP), Ribeirão Preto, Brazil; ^4^Experimental Research Unit (UNIPEX), Botucatu Medical School, São Paulo State University (UNESP), Botucatu, Brazil; ^5^Medicine Department, São Carlos Federal University, São Paulo State, Brazil

## Abstract

Experimental studies have shown the action of green tea in modulating cardiac remodeling. However, the effects of green tea on the cardiac remodeling process induced by doxorubicin (DOX) are not known. Therefore, this study is aimed at evaluating whether green tea extract could attenuate DOX-induced cardiac remodeling, assessed by cardiac morphological and functional changes and associated with the evaluation of different modulators of cardiac remodeling. The animals were divided into four groups: the control group (C), the green tea group (GT), the DOX group (D), and the DOX and green tea group (DGT). Groups C and GT received intraperitoneal sterile saline injections, D and DGT received intraperitoneal injections of DOX, and GT and DGT were fed chow supplemented with green tea extract for 35 days prior to DOX injection. After forty-eight hours, we performed an echocardiogram and euthanasia and collected the materials for analysis. Green tea attenuated DOX-induced cardiotoxicity by increasing cardiac function and decreasing the concentric remodeling. Treatment with DOX increased oxidative stress in the heart, marked by a higher level of lipid hydroperoxide (LH) and lower levels of antioxidant enzymes. Treatment with green tea increased the antioxidant enzymes' activity and decreased the production of LH. Green tea extract increased the expression of Top2-*β* independent of DOX treatment. The activity of ATP synthase, citrate synthase, and complexes I and II decreased with DOX, without the effects of green tea. Both groups that received DOX presented with a lower ratio of P-akt/T-akt and a higher expression of CD45, TNF*α*, and intermediate MMP-2, without the effects of green tea. In conclusion, green tea attenuated cardiac remodeling induced by DOX and was associated with increasing the expression of Top2-*β* and lowering oxidative stress. However, energy metabolism and inflammation probably do not receive the benefits induced by green tea in this model.

## 1. Introduction

Doxorubicin-induced toxicity is a severe cardiooncology problem in children and adults. Doxorubicin (DOX) is a chemotherapeutic drug with a broad spectrum of activity against various hematological and solid tumors [1]. But its adverse effects limit the use of this drug [2]. One of the most critical side effects is acute or chronic cardiotoxicity. The number of cancer survivors has been increasing, which means there is a larger pool of people who are suitable to become cardiac patients [1, 3]. For example, it is estimated that 70% of breast cancer patients will survive at least five years [3].

There are four types of cardiotoxicity caused by DOX: acute, subacute, chronic, and late toxicity [4]. Arrhythmia, ventricular dilation, and diastolic and systolic dysfunction characterize DOX-induced cardiotoxicity [5]. Considering the variety of clinical presentations, it is not easy to precisely specify the incidence and prevalence. But researchers have reported an incidence of lower ejection fraction (<50%) at 9%, within one year after DOX administration [1, 6]. In addition, 32% of adult survivors of childhood cancer had evidence of cardiac dysfunction by global longitudinal strain [7].

Although there has been increasing interest and substantial research conducted regarding DOX-induced cardiotoxicity, a definitive picture of the mechanisms involved in it remains unclear [8]. Studies have suggested that DOX-induced mitochondrial dysfunction is a crucial cause of cardiotoxicity [9]. In addition, the interaction of DOX with iron, generation of reactive oxygen species (ROS), and the binding of DOX with topoisomerase II (Top2-*β*) have proven to be central mechanisms [10]. Therefore, plant-derived polyphenols, well known as rich sources of antioxidants, could have a potential role in preventing or, at least, attenuating the cardiotoxicity in this clinical setting [11].

Green tea (*Camellia sinensis*) is one of the most widely consumed beverages in the world. The common bioactive compounds found in green teas have a polyphenol structure, including flavan-3-ols (catechins), proanthocyanidins (tannins), and flavonols, the biological properties of which are antioxidant, anticancer, antithrombotic, and anti-inflammatory activities [12, 13].

Experimental studies have shown the action of green tea in modulating cardiac remodeling. For example, in the experimental model of myocardial infarction, green tea attenuated cardiac remodeling through the modulation of metabolism, oxidative stress, and apoptosis [14]. But the effects of green tea on the cardiac remodeling process induced by DOX are not known. Therefore, this study is aimed at evaluating whether green tea extract could attenuate DOX-induced cardiac remodeling, as assessed by cardiac morphological and functional changes and associated with the evaluation of different modulators of cardiac remodeling, including Top2-*β*, oxidative stress, energy metabolism, inflammation, and extracellular matrix components.

## 2. Materials and Methods

**Figure 1:**
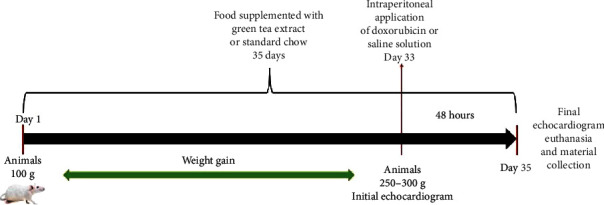
Study development. On day one, we received the animals at approximately 100 g. Since then, they started to received their respective chow. When they reached a weight between 250 g and 300 g, we performed the first echocardiogram and intraperitoneal injection of DOX or saline solution according to which group the animals belonged to. On the thirty-fifth day, at forty-eight hours after administering the DOX or saline solution, we euthanized the animals and collected the blood and cardiac samples for analysis.

The study was approved by The Ethics Committee on Animal Experiments of our institution, and we provided care according to the *Guide for the Care and Use of Laboratory Animals* published by the National Institutes of Health (NIH publication 85–23, revised 1996). We used male Wistar rats weighing between 250 and 300 g, which were acquired from the central animal facility of our institution. The animals were divided into four groups: the control group (C; *n* = 15), which consumed standard chow and received intraperitoneal sterile saline injections; the green tea group (GT; *n* = 15), which consumed food supplemented with green tea extract and received intraperitoneal sterile saline injections; the DOX group (D; *n* = 15), which consumed standard chow and received intraperitoneal injections of DOX; and the DOX/green tea group (DGT; *n* = 15), which consumed food supplemented with green tea extract and received intraperitoneal injections of DOX, and the animals that had food added with green tea received the same for 35 days, prior to DOX injection. The green tea extract used was Polyphenon 60 (Sigma-Aldrich Canadá, Oakville, ON, Canadá). Considering a Wistar rat's feed intake, about 30 g/day, each animal had an intake equivalent to six or seven cups of green tea per day in 60 kg humans [14]. All groups received intraperitoneal injections of DOX or intraperitoneal injections of sterile saline measuring 20 mg/kg weight, respectively. This dose of DOX in rats is equivalent to 250 mg in a 70 kg human [15]. In Figure 1, it is possible to identify how the present study was carried out.

### 2.1. Echocardiography

All animals were submitted to echocardiographic evaluation prior to and forty-eight hours after the DOX injection. The rats were anesthetized with an intramuscular injection of a mixture of ketamine (50 mg/kg) and xylazine (0,5 mg/kg). The apparatus used for evaluation was the HDI 5500 (Philips), equipped with a multifrequency transducer of 7–12 MHz. The examinations were performed by the same examiner (an experienced cardiologist), who did not know which of the previously established groups the animals belonged to. The method was previously set in our group [16, 17].

### 2.2. Morphometric Analysis

Forty-eight hours after the echocardiographic study, we euthanized the animals with a high dose of pentobarbital, dissected their hearts, and collected the blood. Part of the heart was stored in a freezer at −80° C for later analysis. Transverse sections of the left ventricle were fixed in 4% buffered formalin and embedded in paraffin.

### 2.3. Oxidative Stress and Energy Metabolism

We used samples from the left ventricle for protein quantification. Samples of approximately 200 g from the left ventricle were homogenized in sodium phosphate buffer (0,1 M, pH 7,0) and centrifuged at 10,000 rpm for 15 minutes at -4°C, and supernatant helped to determine the concentration of lipid hydroperoxide (LH) and the activity of antioxidant enzymes, because the lipid hydroperoxide in the myocardium served as an oxidation marker of oxidation. We also evaluated the activity of the antioxidant enzymes catalase (CAT), superoxide dismutase (SOD), and glutathione peroxidase (GPx) in the myocardium and determined the concentrations according to previously described methods [18, 19]. On the energetic metabolism, we evaluated the activity of phosphofructokinase (PFK), 3-hydroxyacyl coenzyme A dehydrogenase (3-OHADH), lactate dehydrogenase (LDH), pyruvate dehydrogenase (PDH), and citrate synthase (CS). We also evaluated the activity of the enzymatic complex of the mitochondrial respiratory chain I, complex II, and ATP synthase activity according to the method described above [19, 20].

### 2.4. Western Blot

We extracted left ventricle samples using RIPA buffer for detection of TNF*α*, collagen I and III, AMPK*α*, PPAR*α*, phosphorylated/nonphosphorylated AKT, and Top2-*β*. We then centrifuged the samples at 12,000 rpm at 4°C for 20 minutes and collected the supernatant. We quantified the proteins present in the supernatant using the Bradford method. The SDS-polyacrylamide gel and the protein were transferred to a nitrocellulose membrane. The membrane was blocked with 5% skimmed milk powder in Tris buffer containing 1 M Tris, pH 8,0, 5 M NaCl, and Tween 20 at room temperature for 1 hour 30 minutes. The membranes were incubated with their respective primary antibodies (Table 1) for 12 hours. We then washed the membrane with Tris-buffered saline (TBS) and Tween 20 and incubated it with the peroxidase-conjugated secondary antibody (Table 2) for 1 hour 30 minutes. We also performed the same procedures to determine GAPDH, which we used as a normalizer for the described proteins. With the data generated by the Carestream Molecular Imaging Analyzer (Carestream, Inc., USA), the quantification was performed as follows: we normalized the proteins of interest for one standard sample of one animal and repeated on all the gels; we normalized the GAPDH in the same way by the animal and repeated in all the gels; the proteins (already normalized) were then normalized by the GAPDH (already normalized), to obtain the result of the expression by western blot.

### 2.5. Determination of Cardiac Metalloproteinase Activity: Zymography

We performed the zymography according to the method described previously [21, 22]. Briefly, we used 30 mg of cardiac tissue, added it to an extraction buffer, macerated and centrifuged it, and collected the supernatant. We used the Bradford method to quantify the proteins [23]. We identified three bands corresponding to metalloprotease 2 (MMP-2): inactive MMP-2 (pro-MMP-2), with a molecular weight of approximately 75 kDa, and the active form (active MMP-2), with a molecular weight of approximately 62 kDa, and an intermediate band with a molecular weight 66 kDa [24]. We normalized the quantification of MMP-2 activity by the control animal, which was repeated in all gels [25]. In addition, we determined the activity rate using the proportion of active/inactive bands.

### 2.6. Morphological Study

We dissected the hearts and sectioned the left ventricle at 4 mm from the apex in a 3 mm thick fragment, which was fixed in a 4% buffered formalin solution for 48 hours, according to previous reports [26]. After fixation, we included the tissue in paraffin blocks and obtained coronal sections for posterior histological analysis. We prepared the blades with coronal histological sections and stained them using the Picro Sirius red technique, used specifically for the visualization of collagen. We analyzed 30 to 40 fields per ventricle, excluding perivascular collagen. We made readings using a 40x objective, polarized light, LEICA DM LS microscope, which was coupled to a video camera that sent digital images to a computer equipped with the image analysis program Image-Pro Plus (Media Cybernetics, Silver Spring, Maryland, USA). We calculated the average of the collagen fractional area by the percentage of red color (collagen) per field [26].

### 2.7. Immunohistochemistry

In brief, we dewaxed cardiac tissue in xylene and alcohols, then recovered the antigens using sodium citrate tribasic dihydrate (SIGMA, S4641) in a steamer at 60°C for 20 minutes, followed by peroxidase blockage (3% hydrogen peroxide). Next, we incubated the primary antibody, anti-CD45 (1 : 50, 550539; BD Biosciences), overnight, then incubated it with Histofine Simple Stain Rat MAX PO polymer (MULTI; Nichirei/414191F), which is a combining amino acid polymer with peroxidase (PO) and a secondary antibody, for 1 hour. Then, we applied diamine benzidine chromogen (DAB; Thermo Scientific kit—UltraVision Quanto Detection System; TA-060-HDX) for 30 seconds, followed by a counterstain with hematoxylin (Mayer's hematoxylin solution, SIGMA MHS16-500 ml). We captured digitized images (200 magnification) using a video camera attached to a microscope (Leica Microsystems, Germany). We quantified CD45 in a way similar to the procedure for the collagen fractional area. We measured it by the sum of the percentage of brown cells (CD45) per field [27].

### 2.8. Statistical Analysis

We evaluated the results by comparing groups using a two-way ANOVA. The statistical analysis considered the interaction between the two factors, DOX and green tea, showing a *P* value for this comparison (*P*_i_). If *P*_i_ was less than 0,05, the two-way ANOVA was completed by the Holm-Sidak test to find which group was different from the others.

If there was no interaction among the groups, the statistical test compared both groups treated with DOX (D and DGT) versus the groups not treated with DOX (C and GT), showing a *P* value for the presence of the DOX factor (*P*_d_). The same was stated for the presence or absence of the green tea factor, where GT and DGT were compared with C and D, showing a *P* value (*P*_gt_).

The level of significance was 5%. The variables were presented as a mean ± standard deviation. Data with nonnormal distributions were normalized for comparison.

## 3. Results

We performed an echocardiogram prior to DOX injection to assess whether there was homogeneity among the groups. There were no differences between the groups (data not shown).

Table 3 shows the echocardiographic evaluation after forty-eight hours of treatment with DOX, which decreased the heart rate and increased left atrium (LA). Green tea attenuated DOX-induced remodeling by increasing the heart rate and decreasing LA, posterior wall thickness (PWT), and relative wall thickness (RWT).

Treatment with DOX increased oxidative stress in the heart, marked by a higher level of LH and lower levels of CAT, SOD, and GPx. Treatment with green tea extract (DGT) increased the antioxidant enzymes' activity (SOD, GPx) and decreased the production of LH, as shown in Table 4. Considering energy metabolism, in the D group, the activity of glycolytic enzymes PFK and LDH increased and the activity of 3-OHADH, from FA oxidation, decreased. Green tea attenuated DOX effects on glycolytic and FA oxidation pathways. The activity of ATP synthase, from electron transport chains, decreased in the D group, but green tea could not recover this. DOX reduced the activity of CS, from the citrate cycle and complexes I and II in both groups D and DGT, as shown in Table 5.

**Figure 2:**
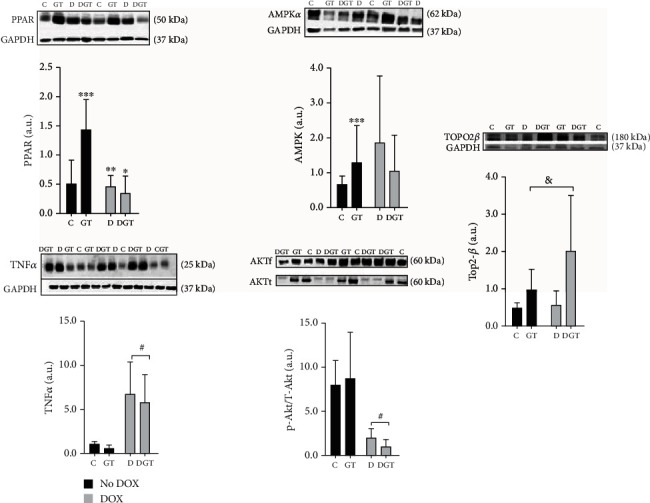
Plot and western blot images showed the expression of proteins in the heart forty-eight hours after injection with doxorubicin (DOX). Groups: C: control; GT: green tea; D: doxorubicin; DGT: doxorubicin+green tea. PPAR*α*: peroxisome proliferator-activated receptor; AMPK*α*: activated protein kinase; TNF*α*: tumor necrosis factor-alpha; P-akt/T-akt: protein kinase B phosphorylated; Top2-*β*: topoisomerase II*β*. *P*_i_: *P* value for interaction between factors GT and DOX. *P*_gt_: *P* value for the differences between the groups with GT and without GT; *P*_d_: *P* value for the differences between the groups with DOX and without DOX. For PPAR*α*: *P*_i_ = 0,001 and AMPK*α*: *P*_i_ = 0, 04, there were interactions between GT and DOX. Thus, for PPAR*α* and AMPK*α*, we considered ^∗^DGT ≠ C, ^∗∗^D ≠ GT, ^∗∗∗^C ≠ GT, and ^∗∗∗∗^D ≠ GT. Comparisons without interaction considered the groups ^#^DOX (D + DGT) ≠ no DOX (C + GT); ^&^GT (GT + DGT) ≠ (C + D). For TNF*α*, *P*_d_ = 0, 01 and for P-akt/T-akt, *P*_d_ = 0, 02. For Top2-*β*, *P*_gt_ = 0, 03.

In relation to proteins analyzed by western blot, green tea extracts increased the expression of AMPK*α* and PPAR*α* in only animals that did not receive DOX. Green tea extracts increased the expression of Top2-*β* independent of DOX treatment. Both groups that received DOX presented with a lower ratio of P-akt/T-akt, a higher expression of TNF*α*, and intermediate activity of MMP-2, regardless of the presence of green tea, as shown in Figure 2 and Table 6.

**Figure 3:**
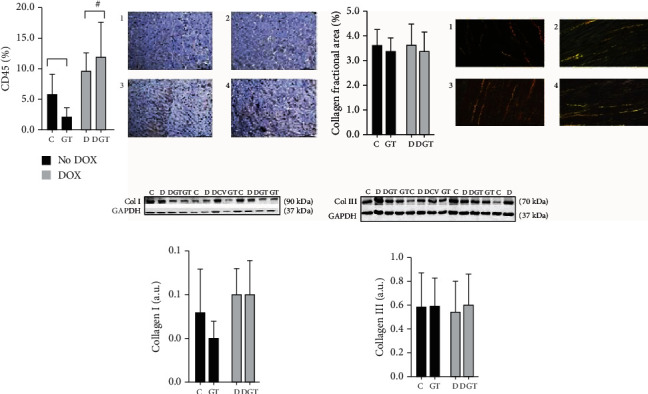
Inflammatory cells and interstitial collagen fraction. Groups: C: control; GT: green tea; D: doxorubicin; DGT: doxorubicin+green tea. In the microscopic images, animal 1 corresponds to group C; 2, GT; 3, DGT; and 4, D. Col I: collagen I; Col III: collagen III; box and bars are expressed in mean ± standard deviation. *P*_i_: *P* value of interaction; *P*_gt_: *P* value of green tea; *P*_d_: *P* value of doxorubicin. After the statistical analysis using two-way ANOVA, no interactions were observed for these data. CD45: ^#^comparisons without interaction considered the differences between: DOX (D + DGT) ≠ no DOX (C + GT): *P*_d_ value < 0,001. Collagen fractional area: Col I, Col III: all the comparisons presented *P* > 0, 05.

In the present study, CD45 expression was higher in animals receiving DOX (D and DGT) when compared with groups that did not receive DOX (C and GT), regardless of the presence of green tea. When analyzed for interstitial collagen and expression of collagen I and III, there was no statistical difference between the groups, as shown in Figure 3.

## 4. Discussion

The aim of this study was to evaluate the effects of green tea on cardiac remodeling induced by DOX. Our data suggest that green tea attenuated the remodeling process. Among the mechanisms involved in DOX-induced cardiotoxicity, we observed that participation of oxidative stress, leading to lipid peroxidation and lowered activity of antioxidant enzymes, which corresponded with higher inflammation, abnormalities in energy metabolism, and cytotoxicity. At least in part, green tea attenuated oxidative stress and cytotoxic damage.

There are different experimental models to study DOX-induced cardiotoxicity. We chose an acute injury pattern, as described previously [25]. In fact, after a few hours of DOX infusion, the presence of inflammation and myocardial damage has already been detected [28]. Electrocardiogram abnormalities have been evidenced within twenty-four hours after DOX infusion in up to 30% of patients [29]. In the present study, after forty-eight hours of DOX infusion, the main findings were an increase in RWT without an increase in ventricular mass, suggesting concentric remodeling and enlargement of LA, which may represent diastolic dysfunction. Both were attenuated by green tea. We also observed lower HR in the DOX group. Despite the fact that systolic dysfunction has been considered a hallmark of DOX-induced cardiotoxicity, the fractional shortening was not affected. However, in the rat model, a lower heart rate may represent systolic dysfunction [30]. Therefore, green tea attenuated morphological changes and improved diastolic and systolic dysfunction as well.

Top2-*β* is a protein that helps DNA fix topological difficulties and protects cells from being destroyed. DOX interacts with DNA and topoisomerase, forming the Top2-DOX-DNA complex, which increases the double-strand breakage, leading to cytotoxic effects [10]. This mechanism is in part responsible for the destruction of cancer cells and the drug's toxicity as well. Regarding the toxicity, the deletion of the Top2-*β* gene protected the heart from damage, confirming that this interaction in part mediates cardiotoxicity [31]. In addition, dexrazoxane, a drug that reduces Top2-*β* levels, protected the heart from cardiac damage [3, 32]. The contradictions here are remarkable, because in general topoisomerase is beneficial to cell survival, but it may also exert a genotoxic role, depending upon the situation. Therefore, the role of topoisomerase levels and its interaction with DOX remains to be elucidated [10]. In the present study, the expression of nuclear Top2-*β* increased in both groups treated with green tea extract. On the other hand, the Top2-*β* expressions were similar in rats treated with DOX when compared with the control group. To our knowledge, this is the first study that investigates the expression of Top2-*β* within the green tea and DOX model. Therefore, our data shows that in the presence of green tea, higher levels of Top2-*β* are not associated with cardiac toxicity. Importantly, green tea has entered as a compound with synergic properties with DOX and other chemotherapy drugs, without increasing cardiotoxicity [33].

Oxidative stress plays a central role in DOX-induced cardiotoxicity. DOX is a substrate for oxireductase enzymes, resulting in a semiquinone radical that binds to iron, resulting in an iron-anthracycline complex that reduces oxygen by forming superoxide. Superoxide, alternatively, has a high affinity with cardiolipin, the main component of the internal membrane of mitochondria that is necessary for oxidative phosphorylation. Cardiolipin is rich in phospholipids and therefore, when interacting with superoxide or DOX, is susceptible to lipoperoxidation. The lipid peroxidation of the inner membrane of mitochondria impairs the transit of ionic transporters, causing damage to energy transfer and increasing the production of ROS. In fact, researchers observed lower concentrations of antioxidants during cardiotoxicity induced by DOX [28, 30, 34]. The present study evidenced higher levels of lipid hydroperoxide (LH), a product of lipoperoxidation, and low levels of antioxidant enzymes induced by DOX. Importantly, green tea reversed those changes. Therefore, we can suggest that the attenuation of oxidative stress was one of the mechanisms involved in the beneficial effects of green tea.

Another potential mechanism involved in the action of green tea on cardiac remodeling is related to cardiac energy metabolism. Under normal conditions, fatty acids are the main substrates mitochondria use to supply energy to the heart. Between 60 and 90% of the heart's ATP is generated by the oxidation of fatty acids, and 10–40% is generated by glucose metabolism. Acetil coenzyme A is the common product of metabolism of FA and glucose, which enters the citrate cycle by generating electron carriers to the electron transport chain from complex I to V in the mitochondrial inner membrane. In complex V, adenosine diphosphate binds to phosphorus (ADP+P) to transform into adenosine triphosphate (ATP) under the action of ATP synthase [35].

In situations of cardiac remodeling, the heart exhibits several changes in energy metabolism, including a fuel preference shift, decreased fuel amount, mitochondrial abnormalities, and impaired transport of energy from mitochondria to the site of utilization [30]. In the present study, the energy metabolism was also involved in the DOX's effects, evidenced by the increased activity of PFK and LDH, suggesting that the anaerobic glycolytic pathway is stimulated. In addition, the decreased activity of 3-OHADH suggests an impairment in FA oxidation. The activity of CS from the citrate cycle, followed by complexes I and II and ATP synthase, was reduced in the D group, but the green tea extract failed to recover this imbalance. Although green tea increased the rate of lipid oxidation, this product did not interfere with the final process of energy production. Therefore, our results suggest that changes in energy metabolism are probably not one of the beneficial effects induced by green tea in this model.

Finally, DOX promotes the release of inflammatory cytokines that are involved in various conditions, including cardiotoxicity. In our study, we observed the elevation of inflammation by the increased activity of intermediate MMP-2, TNF*α* expression, and CD45, which is a common lymphocyte antigen, in both groups treated with DOX. But green tea had no influence on these inflammatory mediators. Therefore, our study did not demonstrate anti-inflammatory effects of green tea, at least with the analyzed variables. While the inflammatory process can produce oxidative stress, oxidative stress induces inflammation, as well. Therefore, they are closely linked, but it is challenging to define which one occurred first. While the inflammatory process can produce oxidative stress, oxidative stress induces inflammation, as well. Therefore, inflammation and oxidative stress are closely linked, but it is challenging to define which one occurred first. It is not possible to explain the lack of green tea anti-inflammatory effects in the presence of an antioxidant attenuation in the present study. We might only hypothesize that green tea might have reduced oxidative stress first, via other reactive oxygen species sources [36].

## 5. Conclusion

In summary, the acute administration of DOX-induced cardiac remodeling, modulated by decreased Top2-*β*, increased oxidative stress and an imbalance in inflammation and energy metabolism. Animals previously fed with green tea extract-enriched chow and treated with DOX presented with greater measurements of cardiac remodeling and function associated with increasing Top2-*β* and lowering oxidative stress. Energy metabolism and anti-inflammation were not some of the beneficial effects induced by green tea in this model, though.

The present experimental study raises the hypothesis of green tea's beneficial effects on preventing cardiac toxicity, which might support future larger research in the clinical setting.

## Figures and Tables

**Table 1:** Concentrations of primary antibodies used for western blot.

Antibody		Dilution (*μ*l)
TNF*α*	Cell Signaling Technology; cs 11948S	1/1000
Collagen I	Santa Cruz, sc 293182	1/100
Collagen III	Santa Cruz, sc 271249	1/100
AMPK*α*	Cell Signaling Technology; cs 2532S	1/1000
PPAR*α*	Santa Cruz; sc-9000 (H-98)	1/100
Phosphorylated AKT	Cell Signaling Technology (Ser473); cs 4060S	1/2000
AKT	Cell Signaling Technology; cs 9272	1/1000
Top2-*β*	Abcam; ab109524	1/10000
GAPDH	Santa Cruz; sc 32233	1/10000

**Table 2:** Secondary antibody concentrations used for western blot.

Analyzed protein	Antibody	Dilution (*μ*l)
TNF*α*	Mouse anti-rabbit sc-2357	1/5000
Collagen I	Mouse anti-rabbit sc-2357	1/2000
Collagen III	Mouse anti-mouse sc-2005	1/1000
AMPK*α*	Mouse anti-rabbit sc-2357	1/5000
PPAR*α*	Mouse anti-mouse sc-2005	1/10000
Phosphorylated AKT	Mouse anti-rabbit sc-2357	1/5000
AKT	Mouse anti-rabbit sc-2357	1/5000
Top2-*β*	Mouse anti-rabbit sc-2357	1/5000
GAPDH	Mouse anti-mouse sc-2005	1/10000

**Table 3:** Echocardiography forty-eight hours after doxorubicin injection.

	C (*n* = 15)	GT (*n* = 15)	D (*n* = 15)	DGT (*n* = 15)	*P*_i_	*P*_gt_	*P*_d_
HR (bpm)	367 ± 9, 50	335 ± 10, 5	290 ± 9, 82	305 ± 9, 82	0,02	0,39	<0,001
LVDD/c (mm)	20, 5 ± 0, 66	19, 9 ± 0, 73	20, 9 ± 0, 67	21, 0 ± 0, 67	0,55	0,69	0,29
LVSD/c (mm)	10, 1 ± 0, 72	9, 26 ± 0, 79	10, 7 ± 0, 74	9, 96 ± 0, 74	0,94	0,28	0,39
PWT/c (mm/g)	4, 39 ± 0, 17	4, 15 ± 0, 19	4, 76 ± 0, 17	3, 97 ± 0, 17	0,01	0,006	0,58
RWT	0, 42 ± 0, 02	0, 42 ± 0, 02	0, 46 ± 0, 02	0, 38 ± 0, 02	0,03	0,08	0,93
LA/c (mm/g)	10, 1 ± 0, 45	11, 1 ± 0, 48	12, 3 ± 0, 45	11, 3 ± 0, 48	0,004	0,93	0,01
LA/AO (mm)	1, 03 ± 0, 05	1, 15 ± 0, 05	1, 14 ± 0, 05	01, 15 ± 0, 05	0,26	0,22	0,27
*A* (cm/s)	78, 0 ± 4, 63	78, 4 ± 4, 88	49, 0 ± 4, 63	68, 4 ± 4, 88	0,05	0,03	<0,001
*E* (cm/s)	88, 1 ± 9, 23	82, 7 ± 4, 46	72, 8 ± 4, 22	74, 2 ± 4, 48	0,44	0,65	0,001
*E*/*A*	1, 18 ± 0, 11	1, 11 ± 0, 12	1, 60 ± 0, 11	1, 09 ± 0, 12	0,07	0,02	0,09
IRTc (m/s)	4, 08 ± 0, 06	3, 99 ± 0, 06	4, 11 ± 0, 06	4, 08 ± 0, 06	0,60	0,30	0,35
SF	0, 51 ± 0, 02	0, 53 ± 0, 03	0, 49 ± 0, 02	0, 53 ± 0, 02	0,72	0,24	0,74
LVMI	1, 75 ± 0, 33	1, 71 ± 0, 39	1, 92 ± 0, 39	1, 72 ± 0, 39	0,37	0,19	0,35

Groups: C: control; GT: green tea; D: doxorubicin; DGT: doxorubicin+green tea. HR: heart rate in beats per minute; LVDD/c: left ventricular diastolic diameter corrected for body weight; LVSD/c: left ventricular systolic diameter corrected for body weight; PWT: posterior wall thickness; LA/c: left atrium diameter corrected for body weight; LA/AO: left atrium diameter corrected for aorta; *A*: wave ratio (represents the peak velocity of transmitral flow during atrial contraction); *E*: wave ratio (represents the peak velocity of early ventricular filling); IRTc: isovolumetric relaxation time; SF: shortening fraction. Values are expressed in mean ± standard deviation. *P* value: two-way ANOVA. *P*_i_: *P* value of interaction; *P*_gt_: *P* value of green tea; *P*_d_: *P* value of doxorubicin. When we observed the interaction between factors doxorubicin and green tea, we considered *P*_i_ < 0, 05. HR: C ≠ GT, C ≠ D, and DGT ≠ D. PWT/c: D ≠ DGT. LA/c: D ≠ C, DGT ≠ D. RWT: D ≠ DGT. Comparisons without interaction: a wave and *E*/*A*: (GT + DGT) ≠ (C + D), *A* and *E* waves: (C + GT) ≠ (D + DGT).

**Table 4:** Cardiac oxidative stress forty-eight hours after doxorubicin injection.

	C (*n* = 8)	GT (*n* = 8)	D (*n* = 6)	DGT (*n* = 6)	*P*_i_	*P*_gt_	*P*_d_
LH (nmol/g of tissue)	206 ± 34, 2	181 ± 6, 30	265 ± 18, 0	166 ± 8, 97	<0,001	0,002	0,22
CAT (*μ*mol/g of tissue)	59, 1 ± 10, 5	37, 4 ± 7, 01	34, 8 ± 7, 18	41, 4 ± 5, 24	0,002	0,30	<0,001
SOD (nmol/g of tissue)	17, 1 ± 1, 40	16, 7 ± 2, 46	13, 4 ± 0, 10	11, 2 ± 2, 82	0,01	0,01	<0,001
GPx (nmol/g of tissue)	27, 6 ± 9, 10	28, 3 ± 1, 72	13, 7 ± 6, 05	20, 5 ± 2, 00	0,01	0,70	<0,001

Groups: C: control; GT: green tea; D: doxorubicin; DGT: doxorubicin+green tea. LH: lipid hydroperoxide; CAT: catalase; SOD: superoxide dismutase; GPx: glutathione peroxidase. Values are expressed in mean ± standard deviation. *P* value: two-way ANOVA. *P*_i_: *P* value of interaction; *P*_gt_: *P* value of green tea; *P*_d_: *P* value of doxorubicin. We observed the interaction between factors in all the variables evidenced by *P*_i_ < 0, 05. LH: D ≠ DGT, D ≠ C. CAT: C ≠ GT, CP ≠ D. SOD: C ≠ GT, C ≠ D, and GT ≠ DGT. GPx: D ≠ DGT, C ≠ D.

**Table 5:** Cardiac energy metabolism forty-eight hours after doxorubicin injection.

	C (*n* = 8)	GT (*n* = 8)	D (*n* = 6)	DGT (*n* = 6)	*P*_i_	*P*_gt_	*P*_d_
PFK (nmol/g tissue)	0, 90 ± 0, 28	1, 28 ± 0, 34	1, 86 ± 0, 31	1, 16 ± 0, 41	<0,001	0,23	0,003
LDH (nmol/mg tissue)	3, 51 ± 0, 25	3, 50 ± 0, 15	7, 69 ± 1, 41	5, 05 ± 0, 51	<0,001	<0,001	<0,001
PDH (nmol/g tissue)	1, 03 ± 0, 21	0, 97 ± 0, 10	1, 03 ± 0, 14	0, 88 ± 0, 13	0,46	0,10	0,42
CS (nmol/mg tissue)	48, 22 ± 8, 58	45, 52 ± 5, 05	29, 05 ± 5, 68	35, 48 ± 6, 22	0,08	0,46	<0,001
3-OHADH (nmol/mg tissue)	20, 91 ± 2, 10	19, 79 ± 2, 74	10, 60 ± 1, 56	18, 89 ± 2, 24	<0,001	0,55	<0,001
Complex I (nmol/mg tissue)	44, 38 ± 5, 97	45, 9 ± 5, 20	34, 2 ± 4, 42	30, 20 ± 6, 14	0,19	0,55	<0,001
Complex II (nmol/mg tissue)	9, 16 ± 1, 97	8, 53 ± 1, 75	4, 80 ± 1, 23	5, 57 ± 0, 88	0,26	0,91	<0,001
ATPs (nmol/mg tissue)	25, 39 ± 3, 00	21, 86 ± 2, 89	16, 55 ± 2, 02	18, 83 ± 5, 15	0,03	0,63	<0,001

Groups: C: control; GT: green tea; D: doxorubicin; DGT: doxorubicin+green tea. PFK: phosphofructokinase; LDH: lactate dehydrogenase; PDH: pyruvate dehydrogenase; CS: citrate synthase; *β*-OHADH: L-3-hydroxyacyl CoA dehydrogenase; complex I (NADH dehydrogenase): nicotinamide adenine nucleotide dehydrogenase; complex II: succinate oxide reductase; ATPs: adenosine triphosphate synthase. Values are expressed in mean ± standard deviation. *P* value: two-way ANOVA. *P*_i_: *P* value of interaction; *P*_gt_: *P* value of green tea; *P*_d_: *P* value of doxorubicin. When we observed the interaction between factors (*P*_i_ < 0, 05), we compared the groups. PFK: C ≠ D, C ≠ GT, and D ≠ DGT; LDH: C ≠ DX, C ≠ GT, and D ≠ DGT; *β*-OHADH: C ≠ D, C ≠ GT, and D ≠ DGT; ATPs: C ≠ D, C ≠ GT. Comparisons without interactions: CS, complex I, complex II: (C + GT) ≠ (D + DGT).

**Table 6:** Matrix metalloproteinase (MMP-2) activation in myocardial tissue forty-eight hours after injection of doxorubicin.

	C (*n* = 6)	GT (*n* = 6)	D (*n* = 6)	DGT (*n* = 6)	*P*_i_	*P*_gt_	*P*_d_
MMP-2at	9412 ± 6060	7690 ± 3939	11413 ± 10306	8468 ± 12171	0,85	0,49	0,68
MMP-2int.	21907 ± 10304	23576 ± 11532	54553 ± 26211	32163 ± 32206	0,16	0,23	0,02
MMP-2inat	35800 ± 21485	34116 ± 32446	48421 ± 29076	48677 ± 32498	0,93	0,95	0,24
MMP-2at/inat	2, 41 ± 0, 62	1, 14 ± 0, 74	1, 94 ± 1, 76	1, 11 ± 1, 80	0,81	0,79	0,72
MMP-2int/inat	0, 89 ± 0, 62	0, 86 ± 0, 43	1, 94 ± 1, 58	0, 57 ± 0, 21	0,09	0,13	0,53

Groups: C: control; GT: green tea; D: doxorubicin; DGT: doxorubicin+green tea. MMP-2a: active matrix metalloproteinase; MMP-2int: intermediary matrix metalloproteinase; MMP-2inat: inactive matrix metalloproteinase. Values are expressed in mean ± standard deviation. *P* value: two-way ANOVA. *P*_i_: *P* value of interaction; *P*_gt_: *P* value of green tea; *P*_d_: *P* value of doxorubicin. When we observed the interaction between factors (*P*_i_ < 0, 05), we compared the groups. MMP-2int: D ≠ C, D ≠ GT, DGT ≠ C, and DGT ≠ GT.

## Data Availability

The data that support this study are available from the corresponding author upon reasonable request.
